# Editorial: The Fertilization Success From the Oocyte’s Perspective

**DOI:** 10.3389/fcell.2021.810420

**Published:** 2021-12-09

**Authors:** Marcela A. Michaut, Joanna M. G. Souza-Fabjan, Rafael A. Fissore

**Affiliations:** ^1^ Instituto de Histología y Embriología (IHEM), Universidad Nacional de Cuyo, CONICET; Facultad de Ciencias Exactas y Naturales, Universidad Nacional de Cuyo, Mendoza, Argentina; ^2^ Faculdade de Veterinária, Universidade Federal Fluminense, Rio de Janeiro, Brazil; ^3^ Department of Veterinary and Animal Sciences, University of Massachusetts, Amherst, Amherst, United States

**Keywords:** oocyte, fertilization, cortical granules, zona pellucida, female infertility, vitrification, RNA sequencing, ovulation

Fertilization is a crucial process in reproductive biology that ends with the birth of a new individual. Despite its importance, many of the molecular mechanisms that underlie fertilization are still poorly understood. The processes and pathways responsible for successful fertilization in different species have been researched for many years, mainly from the sperm’s point of view, probably because the male gamete is easier to obtain. Therefore, the present research topic aimed to collect different research that analyses the fertilization process from the oocyte’s perspective.

For this Research Topic, we sought high-quality research contributions describing novel insights into the biology of the oocyte in different species, including works on *in vivo* and *in vitro* maturation, *in vitro* fertilization, calcium signalling, and cortical reaction. The final topic issue has 13 published chapters (plus a corrigendum) from 73 authors from 11 different countries. Here, we present in Frontiers in Cell and Developmental Biology Journal, a group of reviews, minireviews, and original research articles covering critical factors of oocyte biology, which use different approaches and models, including human, rat, porcine, and seabass.

This Research Topic has four reviews. Rojas et al. focus on the essential molecular mechanisms regulating the biology of cortical granules before and after the gametes interaction, whose function contributes to preventing polyspermy. The authors underline the unanswered questions in this area and emphasize how elucidating new molecular players and steps involved in cortical granule formation and function could result in new strategies for improving fertilization in humans and animals. Gupta explores the function of the four human zona pellucida glycoproteins, including mutations in these genes that cause infertility. This manuscript also reports studies using native/recombinant human zona pellucida proteins and transgenic animals expressing human zona proteins, and highlight possible differences in sperm-zona pellucida binding between mice and humans. Tokmakov et al. describe the most important molecular and cytological advances underlying ovulation using mainly *ex vivo* approaches. This study focuses on oocyte maturation and on follicle rupture steps of ovulation in frogs but draws extensive comparisons with mammals. The review by Chon et al. discusses the current knowledge and future perspectives of premature ovarian insufficiency in women. This condition displays low secretion of ovarian reproductive hormones and a decline in ovarian reserve. The authors emphasize that the incidence of this insufficiency has gradually increased worldwide, its triggers and symptoms are heterogeneous and that a greater number of women endure it without a genetic diagnosis.

Two mini-reviews are part of this Research Topic. Jiménez-Movilla et al. broadly summarize the oocyte molecules implicated in sperm binding and fusion and describe novel methodologies that are making possible the identification of the unknown molecules involved in mammalian fertilization. Rodrigues et al. review the role of germ–somatic cell communication for the formation of primordial follicles, the establishment of the ovarian follicular reserve, and women’s fertility. The authors suggest that overcoming some cases of infertility will require transcriptome studies and characterization of undiscovered genes.

Finally, seven original research articles are part of this collection. Using a rat model of premature ovarian insufficiency Feng et al. investigate the therapeutic effects of the long-acting, PEGylated recombinant human growth hormone (rhGH). The authors show that high dosages of rhGH increased the number of retrieved oocytes, which showed normal energy metabolism. Single-cell transcriptomic analysis of the recovered oocytes showed balanced oxidative stress to cellular oxidant detoxification molecular profiles.

Jia et al. examine how vitrification of pig germinal vesicle (GV) oocytes impact their ability to undergo *in vitro* maturation. The authors used a tandem mass tag-based quantitative approach and bioinformatics analysis to elucidate the proteomic characteristics of vitrified porcine oocytes. The study identified 153 differentially expressed proteins, which adds significantly to our knowledge of how cryopreservation damages oocytes and provide a means to investigate corrective approaches. Using the same cell model, López et al. evaluate how the vitrification of porcine GV oocytes affects early embryo development by monitoring changes in chromatin integrity and actin microfilament distribution. Besides affecting viability and maturation rates, embryos resulting from vitrified GV oocytes displayed the altered distribution of actin microfilaments and chromatin integrity, which explains their low rate of development. Liu H. et al. used porcine cumulus-oocyte complexes (COCs) to investigate during *in vitro* maturation the role of the Calcium-Sensing Receptor (CASR) on cumulus expansion. CASR expression is regulated by follicle-stimulating hormone (FSH), possibly participating in the FSH-stimulated cumulus expansion observed during the resumption of meiosis in this species. Additional studies using pharmacological reagents implicated CASR on the FSH-mediated cumulus expansion, as assessed by changes in morphology and mRNA expression of specific enzymes. Nevertheless, more studies are needed to identify the role of CASR on the cumulus expansion of porcine oocytes.

Izquierdo-Rico et al. extend the analysis of the zona pellucida glycoproteins expressed in eutherian mammals. Intriguingly, the mouse model has been used extensively to study the zona pellucida even though this species expresses only three out of four glycoproteins—ZP1, ZP2, and ZP3–, ZP4 being a pseudogene. The authors performed phylogenetic, molecular, and proteomic analyses to more comprehensively evaluate the status of ZP4. They find that ZP4 pseudogenization is restricted to the subgenus *Mus.* In addition, they performed cross *in vitro* fertilization between rodent species with three and four ZPs and determined that ZPs formed by four glycoproteins are not a barrier for the spermatozoa of species with a ZP formed by three glycoproteins. Moreover, this work identifies several mouse species with four ZPs that can be considered a tool of great value to study the function of ZP glycoproteins in other species, including humans.

Nitric oxide (NO) is a regulatory molecule in the follicular fluid and is a candidate predictor of ovarian response and *in vitro* fertilization outcomes in humans. However, the reported results are inconsistent when investigating the relationship between intrafollicular levels of NO and reproductive parameters. Because of the discrepancies in the reported results, probably due to the instability of NO, Staicu et al. propose using high-performance liquid chromatography (HPLC)-UV to simultaneously measure the oxidation products of NO, nitrite and nitrate. They propose that the oxidation products may be useful in predicting how healthy oocyte donors respond to superovulation and the implantation potential of the embryos produced from their oocytes.

Finally, Liu X. et al. analyzed the molecular signature of distinct cell populations of adult ovaries from Asian seabass using single-cell RNA sequencing (scRNA-seq). Despite the recent use of scRNA-seq analysis to study germ cells or gonadal development, most investigations have focused on a few species such as humans, monkeys, mice, zebrafish, and fly. Less is known about other vertebrates such as fish, which are susceptible to sex reversal (male-to-female). The authors report five distinct cell types in seabass ovaries. These data provide the basis for studying crucial features of germ cells and somatic cells differentiation that will aid in uncovering molecular mechanisms behind gametogenesis and gonad development in fish. In addition, the findings contribute to understand the conservation and divergence in the molecular mechanisms underlying ovarian cells development across Phyla.

Collectively, these papers highlight discoveries that further our understanding of the oocyte and point at areas in need of additional research as we aim to translate the basic knowledge of the biology of the oocyte into novel diagnostic and treatment tools for female infertility.

